# Valorization of Fish Waste Using Biochar and Crude Glycerin as Additives in Composting

**DOI:** 10.1021/acsomega.4c10922

**Published:** 2025-04-30

**Authors:** Brenda K. V. Leite, Ana C. A. Orrico, Marco A. P. Orrico Junior, Rusbel R. Aspilcueta Borquis, Juliana D. Oliveira, Isabelly A. Macena, Erika C. Ota, Ranielle N. S. Vilela, Tarcila S. C. Silva, Luis A. K. A. Inoue

**Affiliations:** † Universidade Estadual do Mato Grosso do Sul, Pós-Graduação em Zootecnia, Aquidauana 79200-000, Brasil; ‡ Universidade Federal da Grande Dourados, Faculdade de Ciências Agrárias, Dourados 79825-070, Brasil; § Universidade Tecnologica Federal do Paraná, Dois Vizinhos, 85.660-000, Brasil; ∥ Embrapa Agropecuária Oeste, Dourados 79804-970, Brasil

## Abstract

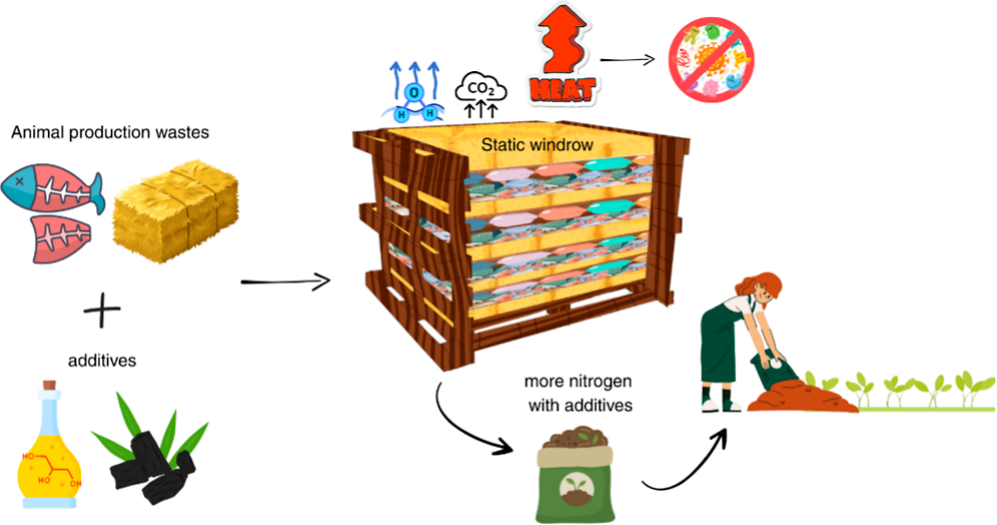

During composting, nitrogen loss primarily occurs in the form of ammonia, which negatively affects the quality of organic fertilizers, because nitrogen is a crucial macronutrient for plant growth. Additives are often employed to mitigate these losses, particularly when composted waste contains high nitrogen levels. This study aims to assess the effectiveness of biochar and crude glycerin as additives in the composting of fish waste in static windrows. Based on fresh weight, five treatments were evaluated: control (no additive), 5 and 10% biochar, and 5 and 10% crude glycerin, over three time periods (50, 70, and 90 days of composting). A 3:1 (mass/mass) ratio of fish waste to bulking agent was used, and the mixture was placed in nylon bags to enhance additive assessment. Thermophilic temperatures were achieved during the early stages of composting and after turning. There were no significant differences (*P* > 0.05) between the control and additive treatments in terms of the reduction in total solids, volatile solids, carbon, hemicellulose, cellulose, and lignin, with averages of 52.0%, 57.8%, 52.3%, 77.3%, 63.9%, and 60.7%, respectively. The additives accelerated fiber degradation (*P* < 0.05). The control treatment exhibited higher nitrogen loss (56.9%) than the biochar treatments (average of 50.6%), whereas the 5% glycerin treatment resulted in the lowest nitrogen loss (26.9%). No significant differences were observed in the macro- and micronutrient concentrations between the treatments (*P* > 0.05). Thus, biochar and crude glycerin are recommended as additives to reduce nitrogen loss without impairing the organic matter degradation.

## Introduction

Fish is a primary source of high-quality animal protein worldwide, and its consumption has risen significantly in recent decades.^1^ In Brazil, a country with vast natural resources and a favorable climate for aquaculture, this trend is reflected in the production of over 860,000 tons of fish in 2022, marking a 2.3% increase compared to that in the previous year.^2^ Nearly half of the fish produced is processed for filleting, with approximately 65% of the total weight potentially becoming waste.^3^ This waste is often repurposed as fish meal and oil for use in animal feed. However, residues that are unsuitable for such uses require proper treatment because of their potential environmental impact.

Fish waste can be efficiently managed through composting, an aerobic degradation process driven by microorganisms that convert organic matter into a stabilized and sanitized final product suitable for use as an organic fertilizer or soil conditioner.^4^ Fish waste is particularly rich in essential nutrients, such as N, P, and Ca, which can be effectively recovered through composting, making it ideal for organic fertilizer production.^5^ However, the high bioavailability and concentration of N present challenges as they promote its loss. Furthermore, the elevated temperatures generated during the degradation of organic matter, along with the alkaline pH, create optimal conditions for nutrient loss, particularly through ammonia volatilization, resulting in reductions of up to 80%.^6^

Biochar, a byproduct of pyrolysis, can play a crucial role in reducing N loss because of its highly reactive surface and microporous structure. These properties allow biochar to retain nutrients, gases, and moisture while enhancing microbial activity, resulting in higher-quality compost with increased N content.^7,8^ In one study, the addition of 10% corn straw biochar cocomposted with layered poultry manure significantly reduced ammonia volatilization, thereby minimizing N loss.^9^ Similarly, incorporating only 5% biochar was sufficient to mitigate N loss during composting of slaughterhouse waste.^10^ The variation in these results may be attributed to the different feedstocks used to produce biochar and the specific characteristics of the composted waste. Identifying the optimal biochar type and dosage for specific waste streams remains a key area for further research and offers opportunities to enhance the efficiency and effectiveness of the composting process.

Crude glycerin, a byproduct of biodiesel production, has shown promising results in terms of N retention during composting. Glycerin is a readily available C source for microorganisms involved in the composting process.^11^ These authors, using crude glycerin in the composting of layer poultry manure, reported that the inclusion of 6% crude glycerin maximized the reduction of total solids (TS) and volatile solids (VS) while minimizing N losses. However, higher inclusion rates can hinder this process because the liquid form of glycerin may create clumps and lead to anaerobic sites. In contrast, the inclusion of 6% glycerin negatively affected mass reduction during the composting of poultry waste, achieving only a 26.8% reduction with no significant impact on N retention.^12^ These differing results may be attributed to variations in the quality of crude glycerin, the composition of which largely depends on the original feedstock and the efficiency of oil extraction in biodiesel production.^13^

In addition to N loss, safety is another critical concern in composting windrows composed of fish waste. These residues, which often include the viscera and blood, can harbor pathogenic microorganisms. To protect workers and minimize health risks, it is advisible to use static windrows (without turning) during the initial phase of composting when the risk is the greatest. This approach is crucial for preventing material exposure to the environment and for reducing the microbiological risks associated with the process.^14^

The role of additives in mitigating N loss during composting is still not fully understood, despite N retention being a critical determinant of compost quality, particularly for fish waste, which is rich in N. This study explored the following hypotheses: (1) biochar and crude glycerin are effective in significantly reducing N loss during the composting of fish waste in static piles, and (2) the dosage of biochar and crude glycerin plays a pivotal role in influencing N retention throughout composting. Accordingly, this study aims to rigorously evaluate the potential of biochar and crude glycerin as strategic additives to enhance N conservation during the composting of fish waste in static systems, offering insights into their practical applications for improving compost quality.

## Materials and Methods

### Site of the Experiment and Characterization of Wastes and Additives

This research was conducted at the Faculty of Agricultural Sciences, Federal University of Grande Dourados, located in Dourados, Brazil (22 11′38″S latitude, 54 55′49″W longitude, at an altitude of 462 m). The climate of the region is classified as Cwa according to the Köppen climate classification system, which corresponds to a humid mesothermal climate characterized by hot summers and dry winters. The fish waste, comprising heads, bones, scales, skin, viscera, and fillets, was provided by a company specializing in the farming and commercialization of fish, located in the municipality of Itaporã–MS.

The material was collected directly from the cold chamber shortly after slaughter and transported to the site where the compost piles were to be prepared. The bulking agent used as a C source was grass hay (Brachiaria brizantha), which was crushed into particles of approximately 2.5 cm in size and mixed with the fish waste in a 3:1 ratio (mass/mass). This ratio was applied to prevent leachate formation and adjust the C/N ratio at the start of the composting process.^15,16^ Biochar was produced from eucalyptus sawdust according to the pyrolysis temperature, heating rate, and residence time.^17^ The crude glycerin had the following composition: 14.2% glycerol, 6.1% methanol, and a chemical O demand of 1532 g O_2_ L^–1^.^13^ The initial characteristics of the materials used are listed in Table 1.

**Table 1:** Chemical Composition of Raw Materials Used in the Composting of Fish Waste in Static Windrows^a^

materials	C (%)	N (%)	C/N	TS (%)	VS (%)	EE (%)	pH
fish	48.1	6.9	7.0	32.5	84.8	27.4	7.8
bulking	52.3	0.5	111.4	90.0	94.2	0.6	7.0
biochar	42.0	NE	NE	96.2	75.7	NE	7.7
glycerin	52.8	NE	NE	96.0	95.0	74.7	4.8

a TS: TS; VS: volatile solids; EE: ether extract; pH: hydrogen potential; NE: not evaluated.

### Treatments and Experiment Conduction

The experiment was designed as a completely randomized trial with split plots over time (50, 70, and 90 days) and five treatment groups. These treatments comprised various concentrations of the additives tested: no additives (control), 5% and 10% biochar, and 5% and 10% crude glycerin, all based on fresh mass. The buried-bag technique was used to ensure precise sampling of the treatments throughout the composting. This method is particularly suitable for high-risk materials that cannot be turned frequently, such as carcass waste.^18,19^ This facilitates a more accurate evaluation of the effects of additives on the material and allows for the monitoring of changes in the chemical composition and degradation over time.^20^

**Figure 1:**
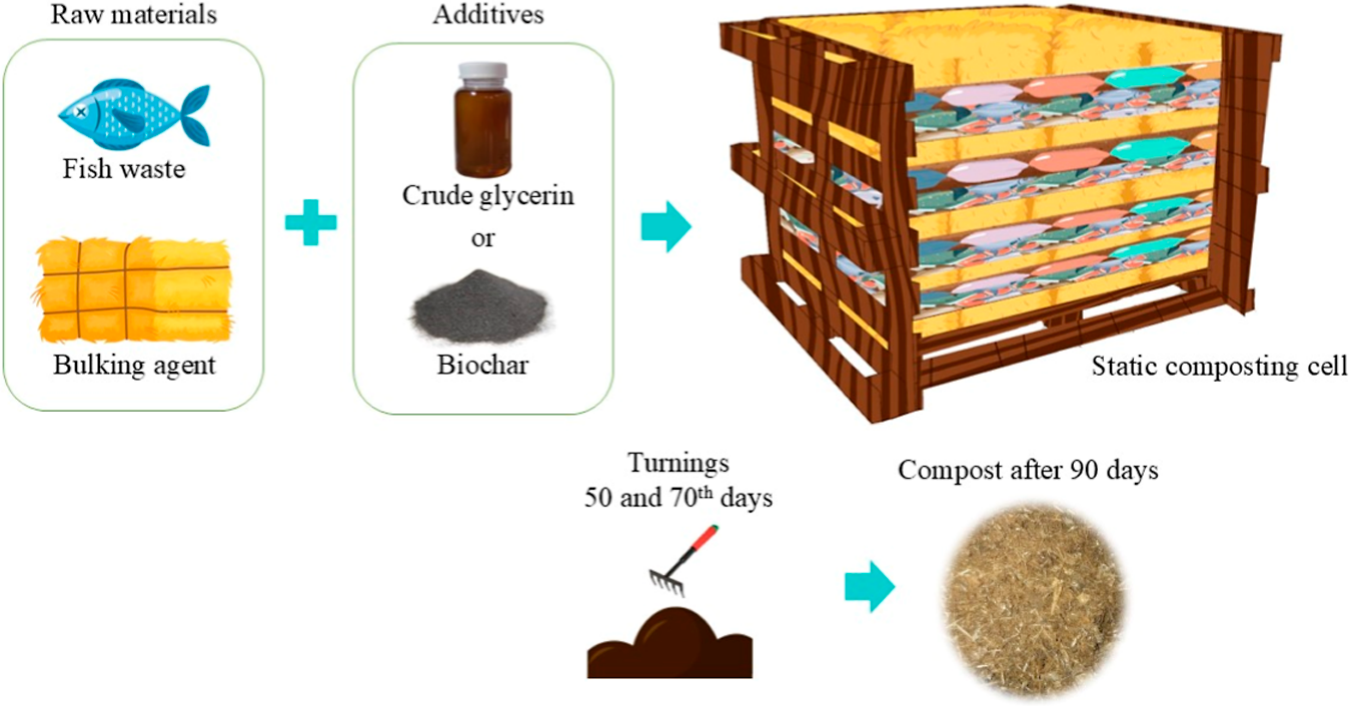
Schematic representation of the experiment.

The bags were constructed from 30 μm nylon mesh,^21^ with dimensions of 25 cm × 35 cm, each with a capacity of approximately 1 kg of fresh substrate. Fifteen bags were used for each treatment, for a total of 75 bags in the experiment. At each sampling interval, five bags from each treatment were removed for analysis and not returned to the piles. For streamlined identification and removal, the bags were color-coded throughout the experiment. Predetermined doses of biochar and crude glycerin were applied to the base material (a mixture of bulking agent and fish waste), which was homogenized and placed in bags before being distributed evenly within the composting piles. Static composting piles were built using wooden pallets spaced to allow natural ventilation and were divided into two composting cells (Figure 1). Each cell measured 1.20 × 0.58 m with a height of 1.00 m. Internally, the piles were lined with Sombrite to prevent material loss through the gaps in the pallets. Five composting cells were used to incubate all of the bags containing the treatments.

The composting cells were filled with alternating layers of the base material (bulking agent) and fish waste with treatment bags randomly distributed between the layers. The first layer comprised the bulking agent, followed by a 10 cm layer of fish waste, after which the bags were placed. This pattern continued until the cells were filled with a top layer comprising a bulking agent. In each cell, four layers of fish waste were formed, ensuring that all treatments were exposed to various conditions at different positions within the piles (base, center, and top).

The experiment lasted 90 days with two turns conducted on days 50 and 70. During the turning process, the material from the composting cells was removed and placed on a plastic canvas for homogenization, sample collection, and moisture content adjustment. This step allowed materials in less favorable positions for degradation to be repositioned closer to the center where the conditions for decomposition were optimal. Samples were collected at various points during the turnings to assess the degradation of the organic compounds and product quality. After turning, the materials were returned to the cells, and the arrangement of the bags was reformed.

Daily temperature measurements were performed inside each pile at 10 randomly distributed points (base, middle, and top) using a probe thermometer. The moisture content was evaluated weekly by measuring the TS in samples collected randomly from different points within the piles. Water was added as necessary to maintain a relative humidity of 40%, 60%, thereby preventing leachate formation.

At the start of the experiment (raw material) and after 50, 70, and 90 days of composting, the pH, TS, VS, organic C, cellulose, hemicellulose, lignin, and N levels were determined and reductions in these parameters were estimated throughout the composting process. After 90 days, the quality of the compost was assessed by analyzing the macromineral (P, K, Ca, Mg, S, and Na) and micromineral (Mn, Fe, Cu, and B) contents.

### Laboratory Analyses

The TS, VS, and pH were analyzed according to the methodology described in.^22^ The ether extract content was determined using the Randall method (INCT-CA G-005/1), as described previously.^23^ The cellulose, hemicellulose, and lignin contents were determined using the methodology proposed in.^24^ The C and N concentrations were determined by using a VARIO MACRO model Elemental Analyzer. The micromineral and macromineral levels were determined using an inductively coupled plasma optical emission spectrometer (PerkinElmer, model Optima 8300, Dual View).^25^

### Statistical Analysis

The treatments were subjected to an analysis of variance. For significant interactions (*P* < 0.05), a split analysis was conducted considering the treatments within each period, with mean comparisons using orthogonal contrasts (C1–control vs additives, C2–biochar vs glycerin, C3–5% biochar vs 10% biochar, and C4 – 5% glycerin vs 10% glycerin). A polynomial regression analysis was performed for the time periods within each treatment. When the interactions were not significant, factors were analyzed independently, with treatments compared using orthogonal contrasts and the time factor managed by polynomial regression.

For the chemical composition analysis of the final compost at 90 days, a completely randomized design with three replicates per treatment was used. The means were compared using orthogonal contrasts (C1, C2, C3, and C4). A principal component analysis (PCA) was conducted to identify the chemical components that defined the treatments. All statistical analyses were performed using R software (version 4.3.2), employing the ExpDes.pt, FactoMineR, and factor extra packages.

## Results and Discussion

**Figure 2:**
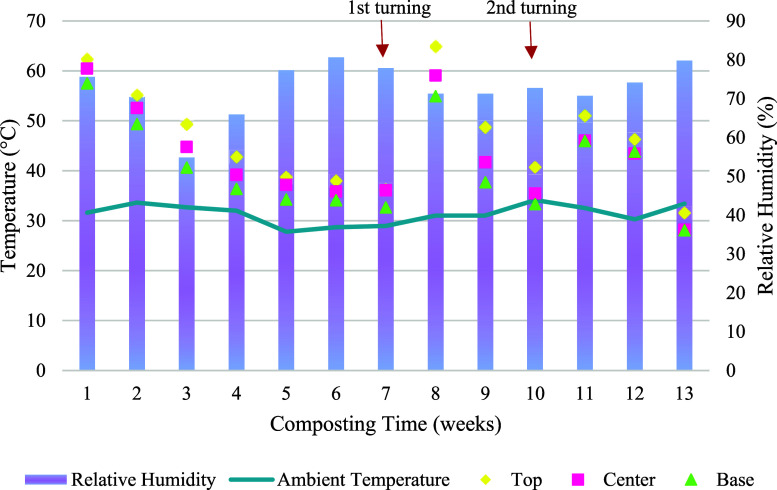
Average weekly air and windrow temperatures and relative air humidity during composting of fish waste in static windrows.

During the first week, the average temperature was close to 60 °C, with peaks of 67.7, 67.1, and 65.0 °C at the top, center, and base of the pile, respectively, on day 2 (Figure 2). The temperature remained in the thermophilic range (>45 °C) until the fourth week and fluctuated near this range between fifth and seventh week. After the first turning and moisture adjustment at the end of the seventh week, the temperatures increased again, reaching a mean of 65 °C at the top of the pile in the eighth week and maintaining thermophilic conditions for another 2 weeks.

Following the second turning at 70 days (10th week), a similar pattern was observed but with a peak temperature of 52 °C. During the last week, the temperature decreased sharply, falling below the ambient temperature, indicating the completion of the composting process, in which the organic compounds were consumed.^6^

Maintaining thermophilic temperatures is crucial for composting animal waste because these residues pose a high biological risk The high biodegradability of fish waste contributed to the rapid microbial activity and heating of the piles.^26^ These temperatures are essential for pathogen inactivation and the efficient degradation of organic matter. Even without turning during the first weeks of composting, there was no excess moisture or leachate formation, suggesting optimal composting conditions despite the higher proportion of fish waste compared with other studies.^27,28^ The openings on the sides of the windrows and high humidity in the air likely contributed to the maintenance of oxygenation throughout the process.

Turning was essential to redistribute fish waste that was less exposed to degradation or compaction, such as at the base of the windrow, and to improve oxygenation and moisture content. Although small amounts of water were added, the static condition of the piles made uniform water distribution challenging, leading to selective infiltration into less-compacted zones.^10^ Following turnings, the increased availability of water stimulated microbial activity, which led to higher temperatures.^29^ Similar results were reported,^30^ who observed that the temperature increased immediately after turning in a static pile composting system.

The addition of biochar and crude glycerin did not significantly affect the reductions in TS, VS, and C compared to the control (*P* > 0.05) during composting (Table 2). At 90 days, the reductions in these constituents were 52.0%, 57.8%, 52.3%, and 77.3%, respectively. The absence of significant differences between the control and additives suggests that neither biochar nor glycerin impedes microbial activity or the degradation of organic matter.^10^ The high biodegradability of fish waste combined with thermophilic temperatures facilitates intense degradation in the early stages of composting. A mass reduction of approximately 50% was observed, which is consistent with the expectations for composting materials with high organic content.^12^

**Table 2:** Reductions of Total Solids, Volatiles Solids, Carbon, and Nitrogen during the Composting of Fish Waste in Static Windrows, with Two Additives (Biochar and Glycerin) and Two Doses (5% and 10%), at 50, 70, and 90 days of Composting^a^

Composting time (days)	C1			C2			C3			C4		
	control	additives	*P*-value	biochar	glycerin	*P*-value	biochar 5%	biochar 10%	*P*-value	glycerin 5%	glycerin 10%	*P*-value
Total Solid Reduction (%)												
50	32.7	29.3	0.26	29.6	29.1	0.91	29.6	29.5	0.93	25.3	32.9	0.01
70	41.9	39.9	0.64	40.0	39.8	0.92	41.8	38.1	0.29	37.4	42.2	0.04
90	51.7	52.4	0.74	52.3	52.4	0.91	53.1	51.4	0.57	50.0	54.8	0.04
Volatile Solid Reduction (%)												
50	38.4	34.1	0.73	36.9	31.3	0.53	33.1	26.5	0.26	40.6	36.1	0.32
70	45.3	40.7	0.69	41.2	40.2	0.91	43.6	38.5	0.38	38.8	42.0	0.62
90	56.1	59.5	0.38	56.0	62.9	0.39	58.3	53.7	0.21	51.6	55.3	0.51
Carbon Reduction (%)												
50	29.9	25.5	0.37	29.0	21.9	0.07	23.6	34.5	0.03	21.0	22.8	0.91
70	42.3	37.0	0.33	38.3	35.7	0.42	42.5	34.2	0.03	36.5	35.0	0.89
90	52.9	51.8	0.92	53.9	49.6	0.39	58.0	49.9	0.04	48.4	50.8	0.89
Nitrogen Reduction (%)												
50	30.8	22.3	0.01	24.7	19.8	0.03	19.6	29.7	0.01	20.6	19.0	0.72
70	36.7	29.4	0.01	31.7	27.1	0.03	29.1	34.2	0.03	27.3	48.8	0.00
90	56.9	50.5	0.01	51.5	49.6	0.67	50.8	52.1	0.86	26.9	50.4	0.00

a C1, control vs additives; C2, biochar vs glycerin; C3, biochar 5% vs biochar 10%; C4, glycerin 5% vs glycerin 10%. Means followed by different letters in the rows differ according to the Tukey test (*P* < 0.05).

In our study, crude glycerin doses did not have an adverse effect on composting, showing a similar reduction across doses (*P* > 0.05, Table 2). Previous studies have reported that the use of crude glycerin in the composting process increased microbial activity at lower concentrations, while higher doses led to anaerobic conditions that reduced oxygen availability and microbial efficiency.^11,30^ In poultry litter composting with 6% glycerin, a 76% reduction in VS was observed, with decreasing efficiency as glycerin levels increased.^11^ The authors reported that the low moisture content of glycerin and its liquid form tended to promote clump formation, thereby creating anaerobic sites that reduced the aeration capacity of the composting windrows.

The VS reduction was not significantly influenced (*P* > 0.05, Table 2) by the addition of biochar, possibly because of the proportions added to the substrates and the recalcitrant nature of the biochar, which hinders microbial degradation.^31^ Previous studies^32^ also reported lower rates of organic matter degradation with increasing levels of biochar, which supports these findings. Similarly,^32^ no significant increase was observed in organic matter degradation with the use of biochar; however, there was a 20% reduction in the composting time. This acceleration was attributed to the high porosity of the biochar, which improved the aeration and water retention conditions, thereby promoting greater microbial activity.

Mass reduction reflects the use of labile C as a source of microbial energy and the loss of C in the form of aqueous CO_2_. These reductions occur primarily at the beginning of the composting process when there is an accelerated activity of microorganisms, which utilize the available carbon for the synthesis of polymerized compounds that will prevail at the end of the process.^29^ Regarding the reduction in C, a difference (*P* < 0.05) was observed only between the doses of biochar, with the lowest reduction observed at the 10% concentration. This is because biochar is rich in recalcitrant C, which makes its degradation during composting difficult.^31^ The amount of carbon decreases until the composting process reaches stability as carbon is continuously utilized by microorganisms. On the other hand, biochar can preserve carbon due to its absorption capacity.^32^

Studies suggest that the addition of easily degradable C in the form of crude glycerin, intended to adjust the C/N ratio and synchronize the degradation of organic matter,^12^ does not significantly affect C concentrations at the end of composting, likely because microorganisms prefer easily degradable glycerin to the more resistant bulky material. However, at a concentration of 6%, the authors observed a reduced rate of organic matter degradation of approximately 40%. A reduction in carbon of approximately 60% was observed with the addition of 6% CG. Beyond this inclusion, the reductions decreased as the study was conducted exclusively with poultry waste and without a bulking agent, which could further compromise the aeration of the windrows and hinder carbon oxidation, leading to greater carbon degradation at lower levels.^11^

**Figure 3:**
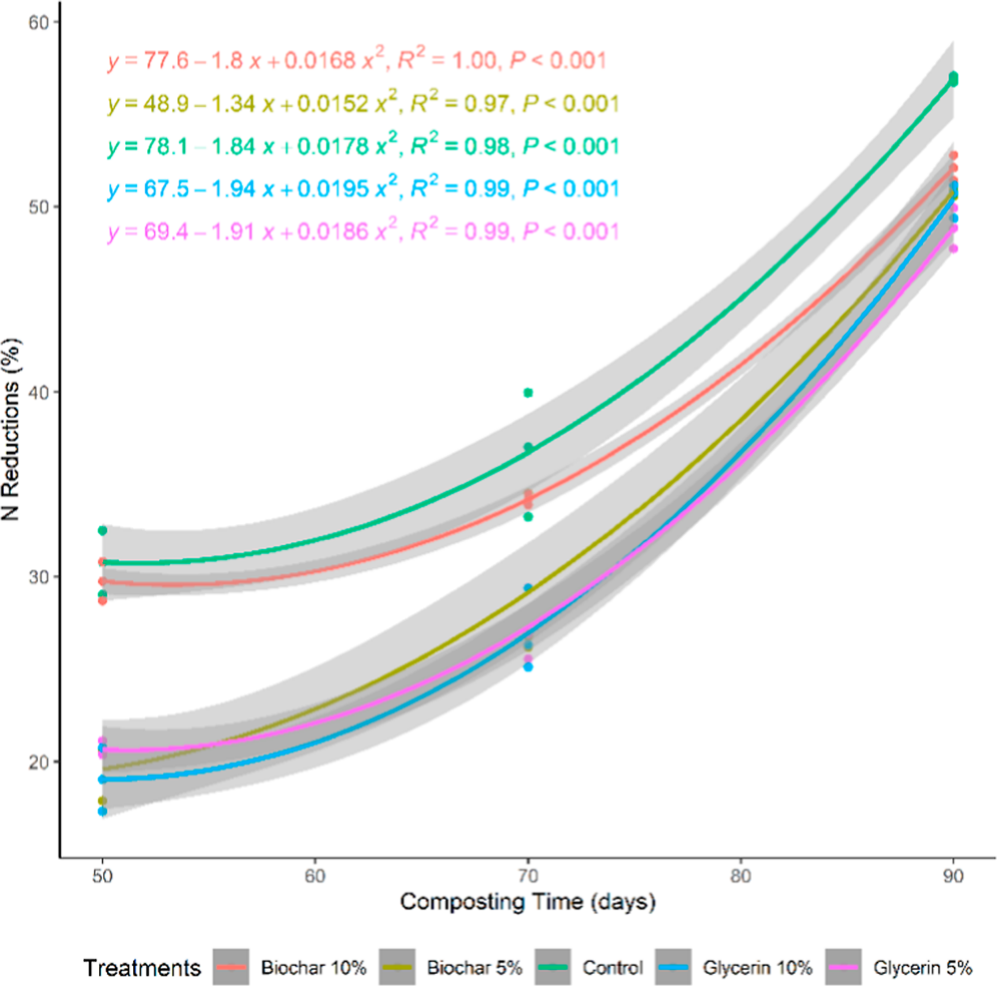
N reduction during the composting of fish waste in static windrows, with biochar or crude glycerin.

The cumulative N losses throughout the composting process indicated that the control (no additives) exhibited higher N losses than the treatments with additives (*P* < 0.05, Figure 3), justifying the use of orthogonal contrasts for analysis (Table 2).

In all periods evaluated (50, 70, and 90 days), the additives were effective in reducing N losses (*P* < 0.05, Table 2), even in materials containing higher amounts of readily available N, such as fish waste. This supports the hypothesis that both additives are effective in mitigating N losses, a result consistent with those of other studies involving high available N levels in cattle slaughterhouse waste.^10^ N loss during composting can be mainly attributed to NH_3_ volatilization, particularly in the early stages, owing to the higher organic matter content available for degradation.^33^

The biochar used in this study demonstrated its efficacy in reducing NH_3_ volatilization (Figure 3), as observed in other composting studies, where biochar reduced NH_3_ emissions by up to 60% when 10% biochar was added to poultry litter compost.^17^ The adsorption of NH_4_^+^ and NH_3_ onto the porous surface of biochar, which can also trap other forms of N and greenhouse gases, likely contributes to this effect, preventing gaseous losses and retaining N in the compost.^7,8^

Gas emissions and N losses are influenced by the thermophilic temperatures reached during composting and can also increase after turning events. However, biochar is also effective in retaining N, even under conditions of high emissions, as reported.^32,34^ In our study, this pronounced N loss behavior after turning events was observed as the windrows remained static until day 50 and handling during turning periods led to accelerated degradation of the remaining organic matter. The authors^32,34^ suggested that higher biochar inclusions were more effective in retaining NH_3_. However, in the present study, no differences were found between the doses at the end of composting (Table 2). Even at lower inclusions, the use of biochar is promising for mitigating NH_3_ volatilization.^35^

Compared with biochar, glycerin was more effective in N retention during the 50- and 70 day evaluations (*P* < 0.05, Table 2). However, by the end of the composting, no significant differences were observed between the two additives (*P* > 0.05, Table 3). Reducing N loss is essential for improving the quality of the resulting compost and achieving a more efficient nutrient recycling process. Accordingly, with the addition of glycerin to layer manure,^11^ there was a 30% reduction in N loss compared to manure without glycerin, using a maximum dose of 12%. However, the 6% glycerin dose recommended by the authors resulted in a higher N content.

**Table 3:** Reductions of Hemicellulose, Cellulose, and Lignin during the Composting of Fish Waste in Static Windrows, with Two Additives (Biochar and Glycerin) and Two Doses (5% and 10%), at 50, 70, and 90 Days of Composting^a^

composting time (days)	C1			C2			C3			C4		
	control	additives	*P*-value	biochar	glycerin	*P*-value	biochar 5%	biochar 10%	*P*-value	glycerin 5%	glycerin 10%	*P*-value
Hemicellulose Reduction (%)												
50	51.7	42.2	0.89	47.2	37.2	0.03	45.6	48.7	0.83	32.7	41.8	0.08
70	60.8	63.9	0.85	66.8	61.0	0.71	68.7	64.9	0.79	54.5	67.4	0.01
90	76.9	77.5	0.91	79.4	75.7	0.69	75.7	83.0	0.18	76.8	74.7	0.73
Cellulose Reduction (%)												
50	18.2	37.1	0.00	30.4	43.7	0.00	27.5	33.2	0.48	37.6	49.8	0.00
70	54.9	54.6	0.98	50.7	58.5	0.83	48.2	53.1	0.41	52.4	64.6	0.00
90	62.3	65.5	0.88	62.4	68.6	0.68	59.6	65.2	0.48	62.7	74.4	0.00
Lignin Reduction (%)												
50	19.9	30.8	0.00	26.4	35.3	0.01	23.7	29.1	0.48	36.4	34.2	0.75
70	48.9	56.0	0.03	52.5	59.5	0.03	46.8	58.1	0.00	52.1	66.9	0.00
90	60.0	61.3	0.82	57.9	64.6	0.04	55.7	60.2	0.52	60.5	68.7	0.01

a C1, control vs additives; C2, biochar vs glycerin; C3, biochar 5% vs biochar 10%; C4, glycerin 5% vs glycerin 10%. Means followed by different letters in the rows differ each other by the Tukey test (*P* < 0.05).

The use of crude glycerin is justified by the addition of labile carbon to a nitrogen-rich source, allowing for the adjustment of the C/N ratio and the synchronization of the decomposition rate of organic constituents.^11^ The microorganisms responsible for the composting process require a C/N ratio of 25–30 for proper metabolism.^29^ The bulky material commonly used in composting contains carbon, which is less available to microorganisms, which can lead to an excess of nitrogen, resulting in its loss through volatilization as NH_3_.

No differences were found^12^ in the N content with the maximum addition of 6% glycerin to poultry production waste. Similar results were obtained with the^12,36^ maximum addition of 6% glycerin, showing no difference in N reduction when using carcasses or solid swine manure. The addition of 5% and 10% glycerin to cattle slaughterhouse waste reduced N loss; no differences were observed between the doses,^10^ corroborating our results (*P* < 0.05, Table 2).

In relation to the reduction in fibers, hemicellulose was significantly influenced (*P* < 0.05, Table 2) when comparing additives and glycerin doses at 50 and 70 days, respectively. Hemicellulose is a cell wall component that is easily degraded during composting and serves as an energy substrate for microorganisms immediately after the consumption of bioavailable nutrients.^32^ The reductions in cellulose and lignin were influenced by the presence of additives at 50 and 70 days (*P* < 0.05, Table 3). A previous study demonstrated that the addition of 15% biochar enhanced the degradation of these components during composting of cattle manure.^32^ This degradation primarily occurs during the thermophilic phase, in which actinomycetes and thermophilic fungi play crucial roles.^32^ Peak activity and enzyme secretion by the fungi occurred between 30 and 60 days of composting.^37^ However, by 90 days in the present study, no significant differences were observed between the additives and the control (*P* > 0.05), indicating that biochar and glycerin primarily accelerated the initial fiber degradation until stabilization was achieved.

Although no significant difference was observed in the reduction of total carbon with the addition of 5% or 10% crude glycerin as an additive, there was a positive effect on the degradation of cellulose and lignin fractions with increasing glycerin levels. When analyzing the degradation of these fractions over time (Table 3), it becomes evident that the reductions intensify with longer composting periods, which may be attributed to two factors. The higher inclusion of crude glycerin (10%) in fish waste composting may have benefited fiber-degrading microorganisms, particularly due to the extended thermophilic phase and the activity of thermophilic bacteria and fungi. Additionally, intense degradation of this fraction still occurred after this phase, possibly driven by microorganisms with an affinity for fibrous materials, especially fungi, which perform better under mesophilic temperatures.

**Figure 4:**
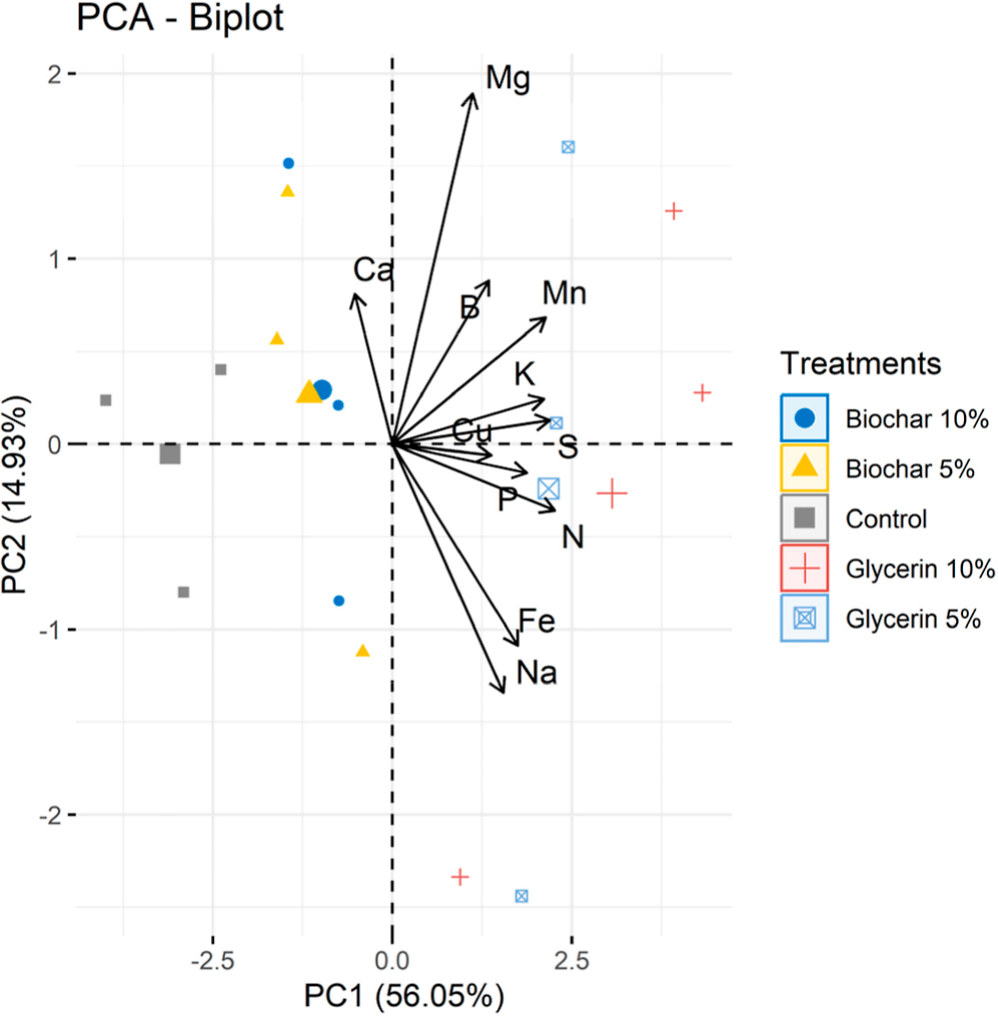
PCA biplot for the quality of compost resulting from fish waste using biochar and crude glycerin as additives.

The use of additives during composting can improve compost quality and contribute to sustainable nutrient recycling in agriculture.^5,38^ At the end of composting (90 days), there were no significant differences in the concentrations of macronutrients and micronutrients between the control and additive treatments (*P* > 0.05, Table 4). PCA (Figure 4) showed that conditions containing crude glycerin influenced the nutrient profile, particularly Na, likely because of its origin as a byproduct of biodiesel production, where Na hydroxide was used as a catalyst in the process.^39^ Conversely,^10^ it was found that biochar had a greater influence than glycerin on slaughterhouse waste.

**Table 4:** Composition of Macronutrients and Micronutrients in the Compost Generated from the Composting of Fish Waste in Static Windrows, with Biochar or Crude Glycerin^a^

nutrients	C1			C2			C3			C4		
	control	additives	*P*-value	biochar	glycerin	*P*-value	biochar 5%	biochar 10%	*P*-value	glycerin 5%	glycerin 10%	*P*-value
P (g/kg)	25.5	27.1	0.00	26.6	27.5	0.05	27.0	26.2	0.25	27.2	27.9	0.27
K (g/kg)	10.3	11.6	0.00	11.2	12.0	0.01	11.1	11.3	0.52	11.8	12.3	0.25
Ca (g/kg)	44.6	45.1	0.27	45.6	44.5	0.01	45.6	45.6	0.97	44.3	44.7	0.42
Mg (g/kg)	2.9	2.9	0.39	2.9	3.0	0.64	2.9	3.0	0.64	2.9	3.0	0.48
S (g/kg)	3.5	3.6	0.01	3.5	3.7	0.00	3.5	3.6	0.34	3.7	3.7	0.85
Mn (mg/kg)	158.2	167.5	0.03	160.9	174.2	0.00	161.3	160.5	0.88	173.3	175.0	0.73
Fe (mg/kg)	935.3	971.4	0.04	950.9	992.0	0.01	947.6	954.2	0.74	980.0	1003.9	0.25
Cu (mg/kg)	13.1	13.9	0.05	13.6	14.1	0.13	13.5	13.7	0.55	14.5	13.8	0.14
B (mg/kg)	7.7	7.9	0.30	7.7	8.0	0.04	7.8	7.7	0.60	8.1	8.0	0.63
Na (mg/kg)	3.6	3.8	0.15	3.7	3.9	0.06	3.7	3.7	0.67	3.8	3.9	0.52

a C1, control vs additives; C2, biochar vs glycerin; C3, biochar 5% vs biochar 10%; C4, glycerin 5% vs glycerin 10%. Means followed by different letters in the rows differ from each other by the Tukey test (*P* < 0.05).

The use of biochar as a quality enhancer has been reported by Kammann et al.,^40^ who have observed its efficiency in gas retention, leading to an increase in the N concentration in the compost, as well as other essential macronutrients for plants, such as P, K, and Ca. Similar findings were reported^41^ when evaluating compost produced from poultry waste with the addition of biochar, where higher levels of Ca and Fe were observed. The ability of biochar to retain water and facilitate cation exchange is an intrinsic benefit that, when applied to composting, contributes to the quality of the organic fertilizer produced. Its application to the soil can positively influence nutrient cycling and reduce losses due to leaching.^10^

Notably, the additives did not worsen the composting process or the concentrations of macronutrients and micronutrients necessary for plant growth. The final compost maintained adequate levels of essential nutrients, making it a viable organic fertilizer for agricultural use. This highlights the importance of research using residues, such as fish waste, as these can provide recycling of nutrients and minerals and how to manipulate these wastes to avoid environmental pollution and human health problems.

## Conclusions

The addition of biochar and crude glycerin to the composting of fish waste effectively reduced the N loss. Both additives, particularly 5% glycerin, enhanced N retention, which is the key to the production of high-quality compost. Importantly, these additives did not hinder organic matter degradation or affect other nutrient concentrations, making them suitable for composting high-N content materials such as fish waste. This study provides valuable insights for optimizing composting processes and supporting nutrient recycling for sustainable agriculture.
